# Effects of dimension reduction of hyperspectral images in skin gross pathology

**DOI:** 10.1111/srt.13270

**Published:** 2023-02-01

**Authors:** Eleni Aloupogianni, Masahiro Ishikawa, Takaya Ichimura, Mei Hamada, Takuo Murakami, Atsushi Sasaki, Koichiro Nakamura, Naoki Kobayashi, Takashi Obi

**Affiliations:** ^1^ Department of Information and Communications Engineering Tokyo Institute of Technology Yokohama Japan; ^2^ Faculty of Health and Medical Care Saitama Medical University Hidaka Campus Hidaka Japan; ^3^ Department of Pathology Faculty of Medicine Saitama Medical University Moroyama Campus Moroyama Japan; ^4^ Department of Dermatology Faculty of Medicine Saitama Medical University Moroyama Campus Moroyama Japan; ^5^ Institute of Innovative Research, Tokyo Institute of Technology Tokyo Japan

**Keywords:** computer‐assisted diagnosis, dimension reduction, gross pathology, hyperspectral imaging, segmentation

## Abstract

**Background:**

Hyperspectral imaging (HSI) is an emerging modality for the gross pathology of the skin. Spectral signatures of HSI could discriminate malignant from benign tissue. Because of inherent redundancies in HSI and in order to facilitate the use of deep‐learning models, dimension reduction is a common preprocessing step. The effects of dimension reduction choice, training scope, and number of retained dimensions have not been evaluated on skin HSI for segmentation tasks.

**Materials and methods:**

An in‐house dataset of HSI signatures from pigmented skin lesions was prepared and labeled with histology. Eleven different dimension reduction methods were used as preprocessing for tumor margin detection with support vector machines. Cluster‐wise principal component analysis (ClusterPCA), a new variant of PCA, was proposed. The scope of application for dimension reduction was also investigated.

**Results:**

The components produced by ClusterPCA show good agreement with the expected optical properties of skin chromophores. Random forest importance performed best during classification. However, all methods suffered from low sensitivity and generalization.

**Conclusion:**

Investigation of more complex reduction and segmentation schemes with emphasis on the nature of HSI and optical properties of the skin is necessary. Insights on dimension reduction for skin tissue could facilitate the development of HSI‐based systems for cancer margin detection at gross level.

## 1 INTRODUCTION

Hyperspectral imaging (HSI) is an emerging modality in medical imaging.^1^, ^2^ Although histopathological diagnosis remains the gold standard for tumor diagnosis, HSI can pave the way toward noninvasive optical biopsy at dermatology and at gross pathology level. Histology is costly and time‐consuming, adding delays in the diagnostic process and increased burden to short‐staffed pathology laboratories.^3^ Applications of HSI for the diagnosis of skin pathologies have been investigated in the past decade in various settings, in vivo, at gross level or during histology.^4^, ^5^ The concentrations of chromophore molecules, mainly melanin and hemoglobin, affect the attenuation of radiation through skin layers.^6^, ^7^ Therefore, spectral signatures of the tissue could give insights about its contents. For example, accelerated angiogenesis or uncontrolled growth of melanocytic cells are both indicators of tumor growth^8^ that result in increased concentrations of hemoglobin and melanin. Under this assumption, tumorous tissue could be detected by its altered spectral signature. Spectral signatures could help measure tissue oxygenation,^9^, ^10^ act as indicators for tumor metabolism,^2^ or detect different skin pathologies.^11^ Differences in the optical properties of the skin can be indicators of cancer growths.^12^, ^13^ Monte Carlo simulations can be used in combination with spectral measurements to take into account the multilayered structure of the skin, in order to provide insight into skin tissue components.^14^, ^15^

HSI systems can capture color information accurately using narrowband filters and do not suffer from metamerism phenomena like RGB images. However, dozen‐ or hundred‐point long spectral signatures increase calculation complexity and harm classification performance.^16^ For this reason, dimension reduction is commonly proposed as a preprocessing step for machine learning with spectral data. It can both reduce classification complexity and discard noise or redundant information from the spectral signature.^17^ Moreover, dimension reduction is frequently used in order to employ machine and deep learning models that are commonly prepared for three‐channel images.

A multitude of dimension reduction techniques for HSI data has been proposed, each with different assumptions and effects. Available methods can be categorized into two broad groups: feature extraction and feature selection. Feature extraction transforms the original data space into a low‐dimensional representation that preserves relationships among samples. Principal component analysis (PCA) is a popular unsupervised feature extractor, that calculates an orthogonal projection so that the few dimensions of the new feature space explain most of the data variance. PCA is commonly applied in medical HSI applications for both classification and reconstruction purposes.^18^, ^19^, ^20^, ^21^, ^22^ Superpixel‐based PCA (SuperPCA) has been proposed as an improvement for hyperspectral image samples that contain inhomogeneous areas^23^ but has not yet been applied on medical HSI. Another unsupervised option is independent component analysis (ICA), which assumes independent components with non‐Gaussian distributions.^24^, ^25^ When pixel labels are available, a supervised equivalent is linear discriminant analysis (LDA).^26^, ^27^ On the other hand, feature selection identifies important wavelength points in the spectrum. Target wavelengths can be selected with random forest importance (RFI)^28^ or sequential forward selection.^29^ Other works have proposed the use of convolutional neural network layers for feature extraction^30^ or recursive feature elimination,^31^ among others.

Samples of skin tissue during gross pathology inherently contain large variability of colors and textures. Compared to other classification/segmentation targets, skin samples pose considerable challenges. Tumor areas are characterized by chaotic, highly inhomogeneous appearance. Spectral signatures may be obscured by hair follicles, inflammation, or tissue dyes. The choice of dimension reduction technique is empirical and depends on the data and the task at hand. Segmentation of medical HSI only recently started capturing research interest.^32^ Until recently, gross pathology research focused only on the classification of entire tissue images or manually selected tissue areas. HSI applications in gross pathology show potential for tasks, such as the detection of tumor margins, identification of disease type, and monitoring of disease progression.^5^ Previous studies investigated the effects of dimension reduction in different multidimensional datasets, including biological data,^33^ general HSI,^34^ or remote sensing HSI.^35^ However, there is a research gap regarding the use of dimension reduction in tasks that utilize medical and/or gross pathology HSI data. The suitability of dimension reduction has not been investigated comparatively in segmentation tasks during skin gross pathology.^5^ Components produced by a suitable dimension reduction method could assist the discrimination between tumor and nonneoplastic tissue. At the same time, the visualization of important components could assist the diagnosis of tumor margins and be more familiar to medical staff. Accurate detection of tumor margins during gross pathology could reduce the need for histological examination. Therefore, duration until diagnosis and associated costs could be greatly reduced.

This study aims to investigate the effect of several standard dimension reduction methods on HSI of raw skin tissue during pathology. The task at hand was tumor margin detection. We designed an HSI acquisition system and prepared an original HSI dataset of ex vivo gross pathology tissue. A variety of standard dimension reduction techniques were applied. An original dimension reduction technique targeting skin HSI was proposed. The advantages and disadvantages of each method are presented, together with quantified results regarding signal preservation. We expect to provide suggestions for preprocessing of skin HSI data in order to facilitate computer‐assisted diagnosis of skin tumors.

## 2 MATERIALS AND METHODS

### 2.1 Imaging system

An original acquisition system for skin tissue with high‐resolution 2D HSI in the visible (VIS) spectral range was designed and implemented. The system consisted of a 2D spectroradiometer (Topcon SR‐5000, 1.4Mpixel CCD image sensor, lens 32 mm, 1376 × 1024 pixel, 380–780 nm), a single‐LED illumination source (SolaX‐iO LE‐9ND55, SERIC), and a pair of polarizers (TS High Contrast Plastic Polarizing Plate, Edmund Optics Japan) in crossed nicols configuration. The target specimen was placed on a porous black‐colored surface, to be easily disinfected. The wavelength range was limited at 420–750 nm, in order to match polarizer response and reduce noisy bands. Integration time was optimized at 618 ms, resulting in an average imaging duration of 3 min. The design and calibration process for the HSI system is described in detail in Ref. [36]. Because of the noncontact imaging process, the reflectance measurements are not affected by pressure phenomena.^37^ The acquisition system was moved inside the pathology laboratory, where pathology experts (TI and MH) performed HSI capture during medical practice. A rectangular region of interest (ROI) to be imaged was manually selected before imaging using a preview tool from the spectroradiometer's software. This way, only the specimen in the ROI was imaged, and unnecessary background was not, thus reducing the capture duration. The largest HSI data cube had spatial dimensions of 500 × 700 pixels and corresponded to a skin sample with a length 8 cm.

Normalized reflectance was recovered from each image pixel as

(1) $$r\;\left(\lambda \right)=\;\frac{I\left(\lambda \right)-I_{b}\left(\lambda \right)}{I_{w}\left(\lambda \right)-I_{b}\left(\lambda \right)}$$

using dark current $I_{b}$ and white reference $I_{w}$ images. Leveraging on the fact that the imaging background had a consistent black color, a mask of tissue pixels was created using iterative clustering and morphological operators. The HSI was converted to a standard RGB image and then transformed to the Lab space. *K*‐means clustering for six color levels was applied three times on the AB channels. Clusters that included border pixels, which were assumed background, were merged and morphological closing was applied. After background removal, only the spectral signatures that correspond to tissue pixels were extracted and used in subsequent experiments.

### 2.2 Experimental dataset

An original HSI dataset of pigmented skin lesions was prepared for this study. The experiments in this study were approved by the Ethical Committee of Saitama Medical University (977), and all participants gave informed consent for the scientific use of their data. HSI capture was integrated into laboratory practice as follows. Pigmented skin lesions diagnosed by expert dermatologists were selected as targets of this study. Target samples after surgical excision were received in the pathology laboratory, where they were immediately imaged with the HSI system, before any preservation process. Tissue was positioned with the surface side up, to simulate in vivo imaging. During gross pathology HSI and because of the static camera setup, image artifacts caused by movement, breathing, and uneven illumination were avoided. Afterward, each tissue sample was sectioned and formalin‐fixed for histology. Due to the fast acquisition time, the HSI process did not cause a significant delay in gross pathology. Two days later, the cross‐section samples were imaged, and their assignment to histology slides was recorded. Histology‐validated labels were constructed by the pathologists, specifically a 2D mask that classified each pixel as inside the tumor margin or not. First, the tumor margins were traced on images of sectioned tissue (TI and MH), using drawing software. These outlines were then used as a reference to create segment masks of tissue margins in the surface tissue HSI data. This way, each HSI data cube was associated with a binary label, derived from histology findings, which assigned each pixel to either to the tumor or to the nonneoplastic tissue, that is, not tumor, class. The final segmentation labels were verified by the pathologists.

A labeled dataset of ex vivo tissue at the surface view (raw dataset) was prepared. The dataset contained 19 HSI, each accompanied by a mask of the tumor margin and the final diagnosis. The dataset contained nine benign samples (melanocytic nevus ×3, intradermal melanocytic nevus ×4, dermatofibroma ×1, melanophage aggregate ×1), a precancer sample (Bowen's disease), and nine malignant samples (basal cell carcinoma ×7, malignant melanoma ×1, mucinous carcinoma ×1). An ROI that contained both tumor and healthy tissue was manually selected for each image of fixed tissue. This resulted in a total of 384 014 spectral signatures, 32% of which were within the tumor margin detected by histology.

### 2.3 Characteristics of skin HSI

The reflectance spectrum of tissue is assumed to be a summation of concentration‐adjusted absorbance terms per each skin chromophore, mainly melanin, oxygenated hemoglobin, deoxygenated hemoglobin, and bilirubin. A high rate of vascularization combined with a large number of melanocytic cells and high hemoglobin and melanin concentrations, respectively, can indicate the presence of malignancy. The narrowband filters of HMSI cameras can isolate the reflectance at each wavelength band and detect peaks that correspond to high concentrations of specific chromophores. Therefore, an obvious choice for dimension reduction is manual selection (MSelect) of wavelengths that correspond to peaks of the chromophore spectra. This corresponds to 540 nm (for hemoglobin) and 650 nm (for melanin) in the case of skin tissue.^38^, ^39^, ^40^

### 2.4 Dimension reduction

**FIGURE 1:**
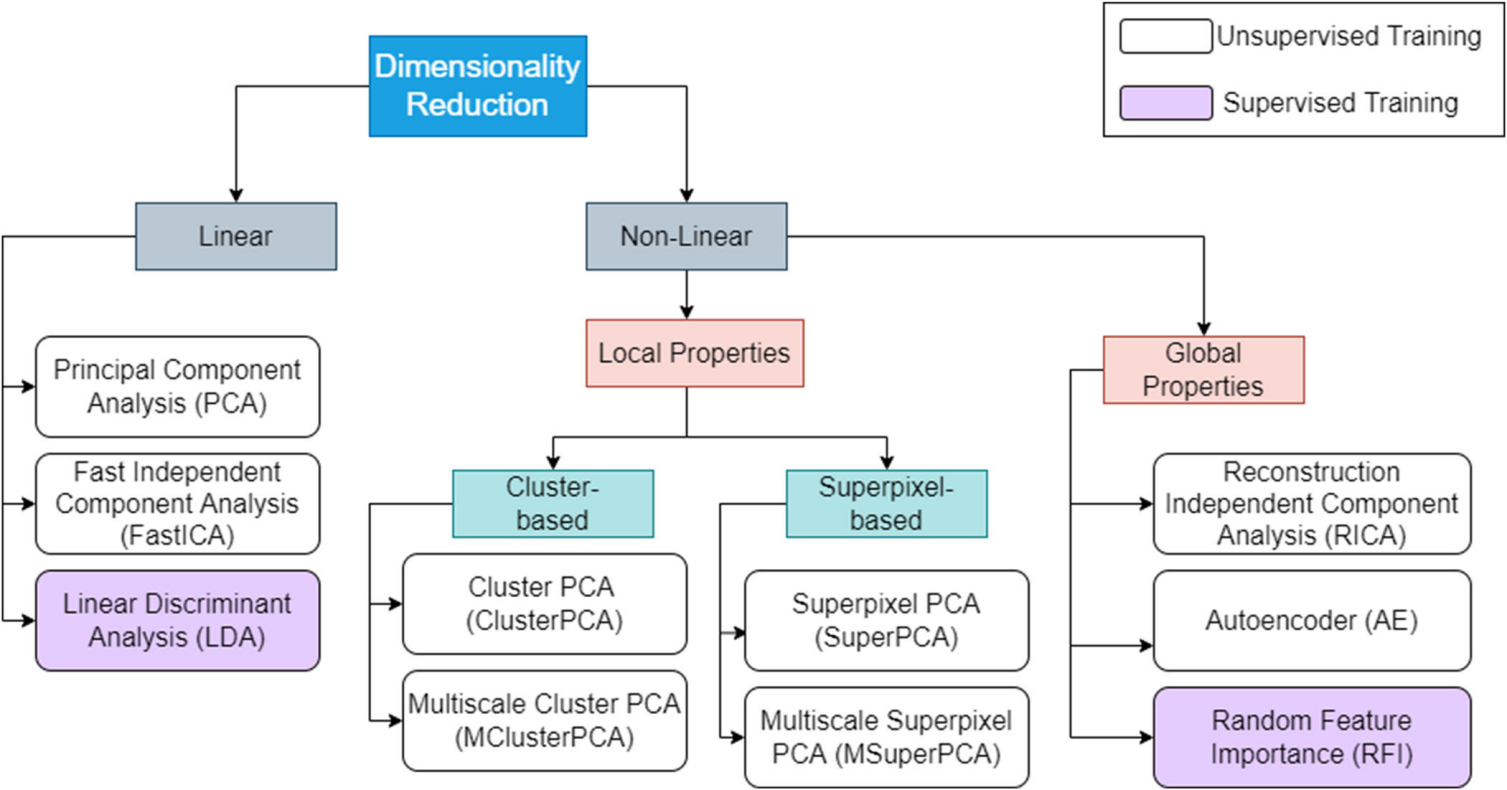
Overview of selected dimension reduction methods

Eleven representative techniques were selected among methods that have been previously proposed for skin tissue or other hyperspectral classification tasks. These methods were compared against a baseline input, which contained the entire 311‐point reflectance spectrum as the feature vector, without any dimension reduction. The selection of methods is shown in Figure 1. Specifically, eight unsupervised and three supervised dimension reduction methods were evaluated. PCA, LDA, and ICA, implemented by FastICA^41^ and by reconstructed ICA (RICA),^42^ are all standard approaches. FastICA is implemented with a set of objective functions that provide speed and reliability. In contrast, RICA uses a reconstruction cost function to learn overcomplete features and performs better on high‐dimensional data. An autoencoder (AE) is a shallow artificial neural network that maps the input to itself, trying to extract image features when learning the mapping process. RFI recovers importance weights for each point of the feature vector, by training an ensemble of weak regressors.

SuperPCA was proposed specifically for HSI classification of remote sensing data.^23^ The HSI data cube is segmented into superpixels, PCA is trained on each superpixel, and individual component scores are returned. Because the superpixel number is an important hyperparameter, a multiscale extension (MSuperPCA) has been proposed. To calculate MSuperPCA, SuperPCA is calculated repeatedly for a set of different superpixel numbers (or scales). Afterward, a classifier is trained on each scale, the results are stacked, and the final class prediction is achieved using a voting scheme. In this work, we apply SuperPCA for the first time on skin HSI.

Building on the knowledge of SuperPCA, we propose cluster‐wise PCA (ClusterPCA). At a macroscopic level, cell and chromophore concentrations on the skin are random. Therefore, the segmentation of a tissue sample into superpixels will contain larger inhomogeneities, compared to buildings and landscapes. Additionally, hair follicles, trauma marks, and diagnostic dyes will affect the variance of the data. Consequently, the coefficients of PCA will be tainted. ClusterPCA solves these problems by incorporating clusters of pixels with spectral similarity, instead of superpixels that are grouped in the spatial dimension.

ClusterPCA is implemented according to the algorithm described in Table 1. The number of clusters is a predefined hyperparameter. For each HSI sample, *n* endmembers are calculated with the N‐FINDR algorithm.^43^ This iterative method identifies “pure” pixels, in the sense of individual substances. Afterward, the spectral similarity of each spectral signature of the HSI against each endmember is calculated by spectral angle mapper (SAM).^44^ A small SAM angle indicates a high similarity of a candidate pixel to the target end‐member. Afterward, cluster labels are identified by assigning a candidate pixel to the cluster of its most similar end‐member. Then, PCA is trained separately on pixels of each cluster, and the transformed scores are extracted. Finally, the ClusterPCA scores are aligned spatially to match the original spatial dimensions of the HSI. ClusterPCA can also be expanded as multiscale ClusterPCA (MClusterPCA), similar to MSuperPCA. In this case, ClusterPCA is calculated repeatedly for a set of end‐member numbers and the classification results for each scale are fused using a voting scheme.

**TABLE 1:** Implementation of cluster‐wise principal component analysis (ClusterPCA)

**Require**: n > 0 endmembers ← N‐FINDR(hsiCube,n) i ← 0 **while** i ≤ n **do** angles_i_ ← SAM(hsiCube, endmembers*_i_*) **end while** labels ← argmin^n^_i = 1_(angles_i_) **while** *i* ≤ n **do** cluster_i_ ← hsiCube(labels = i) scores_i_← PCA(cluster_i_) **end while** scores ← AlignSpatially(scores_1_, …, scores_n_)

Abbreviation: SAM, spectral angle mapper.

### 2.5 Experiments

Dimension reduction can be trained separately on each tissue sample or the entire dataset. The effect of training scope on the eigenvectors and data projections was evaluated on the dataset by PCA, SuperPCA, and ClusterPCA. The transformation vectors corresponding to the three highest eigenvalues were recovered and compared. PCA was implemented in two ways: (a) It was trained on the entire HSI dataset and (b) on each HSI sample individually. For SuperPCA and ClusterPCA, the transformation vectors for one superpixel and one cluster were recovered, respectively. The eigenvectors of PCA were compared with the absorption spectra of skin chromophores, namely, melanin (eumelanin and pheomelanin types), oxygenated hemoglobin (HbO2), and deoxygenated hemoglobin (Hb).^39^, ^40^

After applying dimension reduction, we evaluated binary classification performance with dimension‐reduced datasets. Support vector machine (SVM) with a Radial Basis Function kernel and a 5% outlier rate was employed for classification, because of its good performance in datasets with an unbalanced distribution of classes. A total of 16 HSI were used for training and validation, whereas three HSI were kept in reserve for independent testing. The training was validated with fivefold cross‐validation to calculate average accuracy, sensitivity, and specificity.^45^ All spectra from an HSI sample were included in the same set when splitting data into folds. Each dimension reduction method plus the SVM classifier was trained on four splits and validated on the last. Finally, a classifier was trained on the spectral signatures of the 16 training HSI and evaluated on the reserved test samples. All calculations were implemented in MATLAB 2020b on a system with one CPU (Intel(R) Core(TM) i9‐10900K CPU@3.70 GHz) and one GPU (NVIDIA GeForce RTX 3080).

For all subsequent experiments, dimension reduction methods were trained individually on each image, except for LDA, RFI, and AE, which were trained on the entire dataset. All other methods allow arbitrary selection of the number of retained dimensions, apart from LDA (1 dimension for binary class problem) and MSelect (2 dimensions for the two targeted wavelengths). Because LDA for a binary class problem is sensitive to data noise, spectral signatures were first denoised by keeping several PCA components and LDA was applied afterward (PCA–LDA). The number of kept dimensions was adjusted in the set {5, 10, 20, 50, 100} for eligible methods. SuperPCA was calculated for 20 superpixels and MSuperPCA for values {9, 14, 20, 28, 40} with majority voting. ClusterPCA was calculated for 6 end‐members and MClusterPCA for values {2, 5, 6, 8, 10} with majority voting. RFI was calculated with the gini criterion and AdaBoostM2, with search enabled for all 311 wavelengths. Margin detection was evaluated using the Jaccard coefficient (JacCoef), which for a set of predicted labels *A* and ground truth labels *B* is calculated in the following equation:

(2) $$JacCoef\;=\;\frac{A\;\cap B}{A\;\cup B}$$

Standard metrics for classification performance are provided, specifically accuracy, sensitivity, and specificity. Performance metrics were cross‐validated and presented as a pair of mean value and standard error of mean (SEM).

## 3 RESULTS

### 3.1 Effect of training scope

**FIGURE 2:**
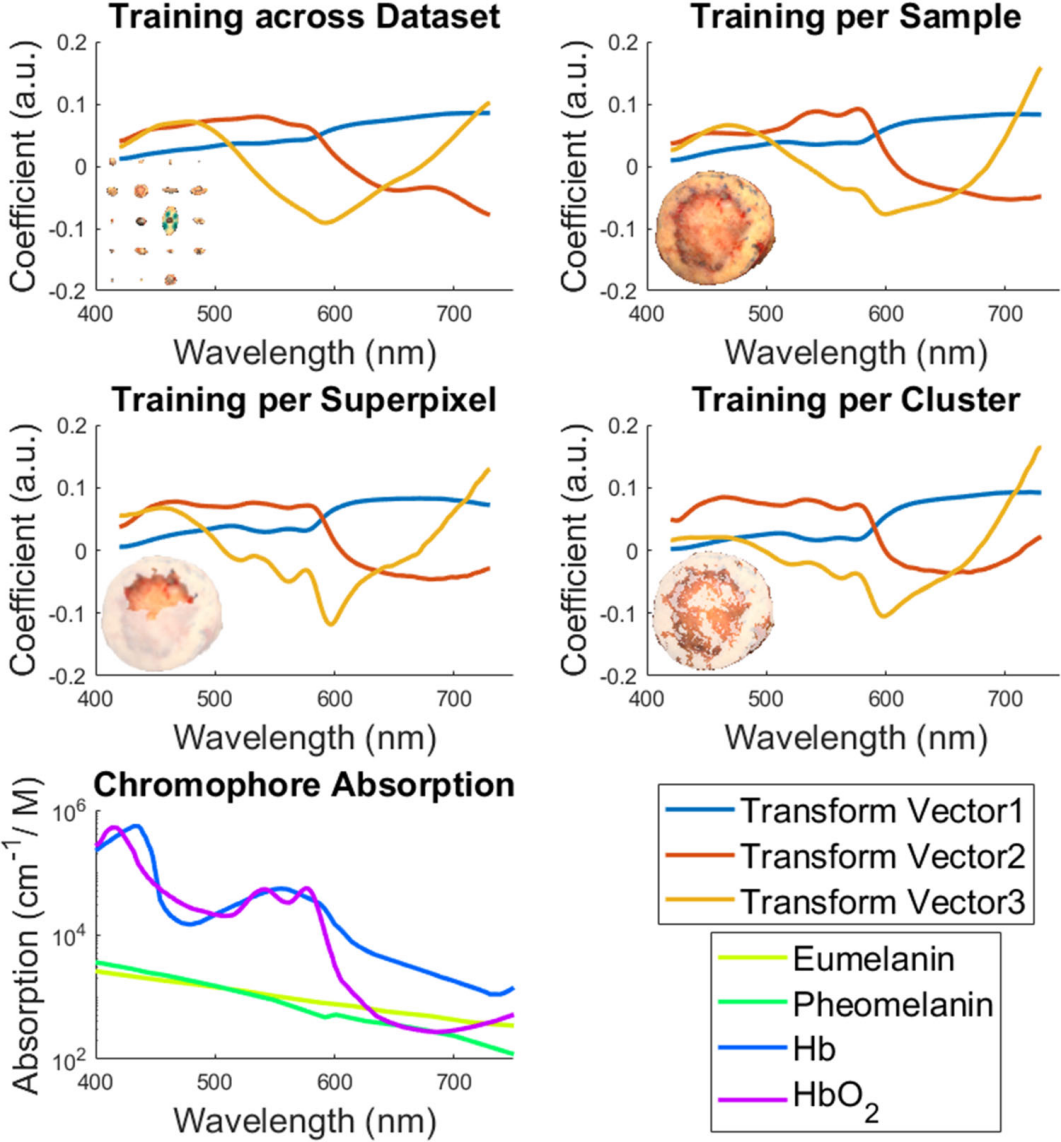
The effect of the scope of principal component analysis (PCA) calculation on transform vector values. Eigenvectors when training PCA on the entire dataset (top left), one sample (top right), one superpixel (middle left), or one cluster (middle right). The training scope is highlighted at the corner of each subgraph. The absorption spectra of skin chromophores (bottom left) are shown for comparison.^39^, ^40^

PCA, SuperPCA, and ClusterPCA were trained on different portions of the same dataset. The eigenvectors are shown in Figure 2. The absorption coefficients of melanin and hemoglobin are shown for comparison. The target HSI tissue sample was diagnosed as basal cell carcinoma, with the interior circular area identified as the tumor margin. Eigenvectors from training on the entire raw tissue dataset are smooth curves along the spectrum. In contrast, PCA trained individually per tissue sample, SuperPCA trained on one superpixel, and ClusterPCA trained on one cluster show more protruding features in the eigenvectors. The first eigenvector is similar for all PCA implementations and shows a sudden slope hike, which coincides with the sudden downward trend of deoxygenated hemoglobin (Hb) at 600 nm. The second and third eigenvectors show twin peaks that match the oxygenated hemoglobin (HbO2) peaks at 542 and 576 nm. Especially for the third eigenvector, the twin peaks are more prominent for SuperPCA and ClusterPCA; therefore, it compresses the hemoglobin component. Additionally, the large valley at 600 nm and the subsequent slope hike coincide with the knee area of the hemoglobin extinction coefficient curve, at which melanin absorption becomes more pronounced, and hemoglobin absorption is minimum.^46^

**FIGURE 3:**
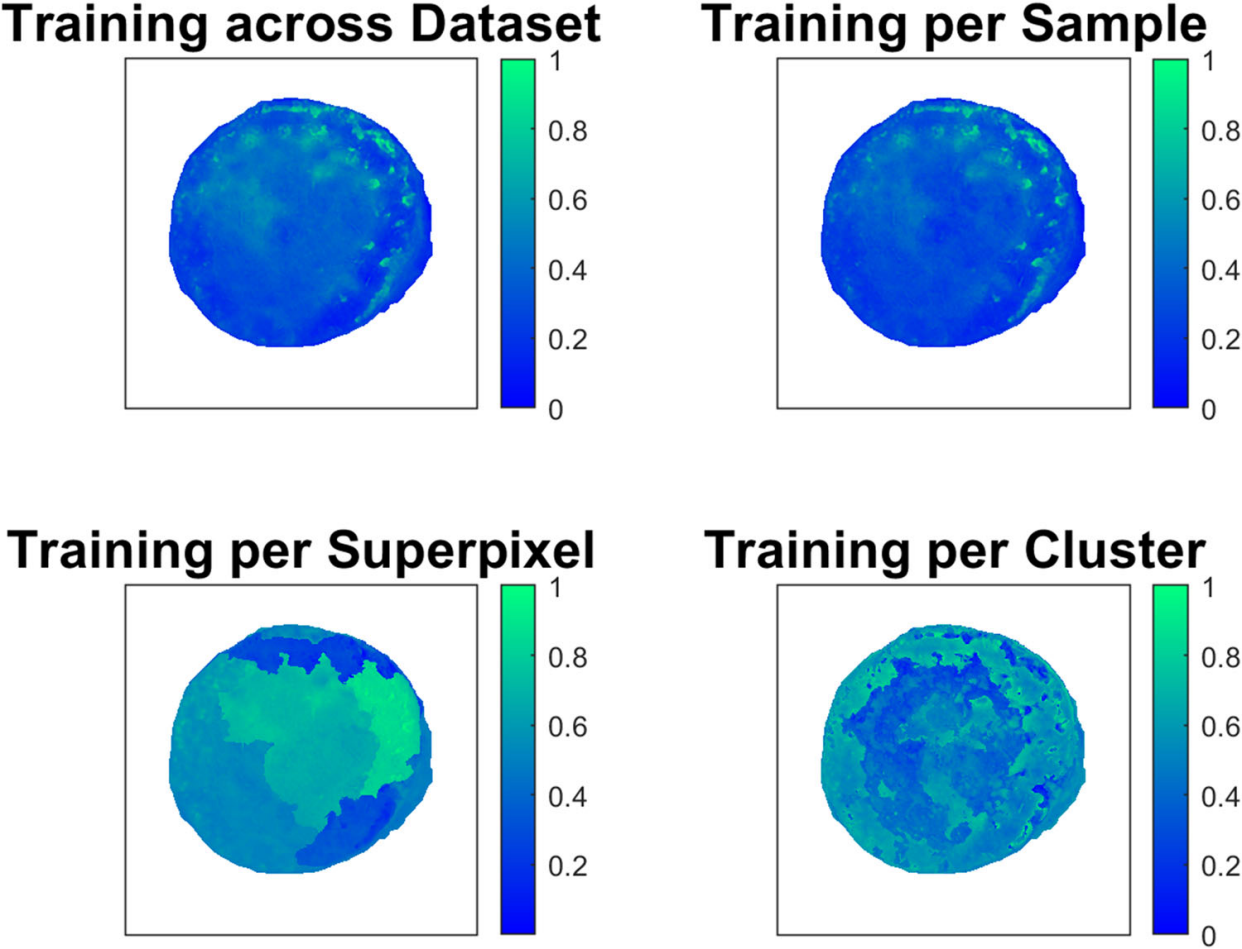
The effect of the scope of principal component analysis (PCA) calculation on the values of the third principal component

In terms of values of the principal components, Figure 3 displays the scores of the third principal component for each training scope. The third component extracts small dot areas in the first quarter of the tissue for dataset‐ and sample‐wide calculation of PCA. These dot areas are actually blood stains that occurred during surgery and are outside the tumor margin, according to the histological evaluation. Furthermore, the scores of SuperPCA retain the approximate spatial boundaries of superpixels, instead of extracting a meaningful tissue component or substance. Therefore, SuperPCA is overtly affected by superpixel segmentation and fails to extract spectral information. In contrast, the third component of ClusterPCA highlights both the middle area of the tissue, which is rich in hemoglobin concentrations and the blood dots on the top right quarter. Therefore, ClusterPCA achieves more effective as well as pathologically interpretable components, compared to the other three applications.

### 3.2 Effect of dimension reduction

**FIGURE 4:**
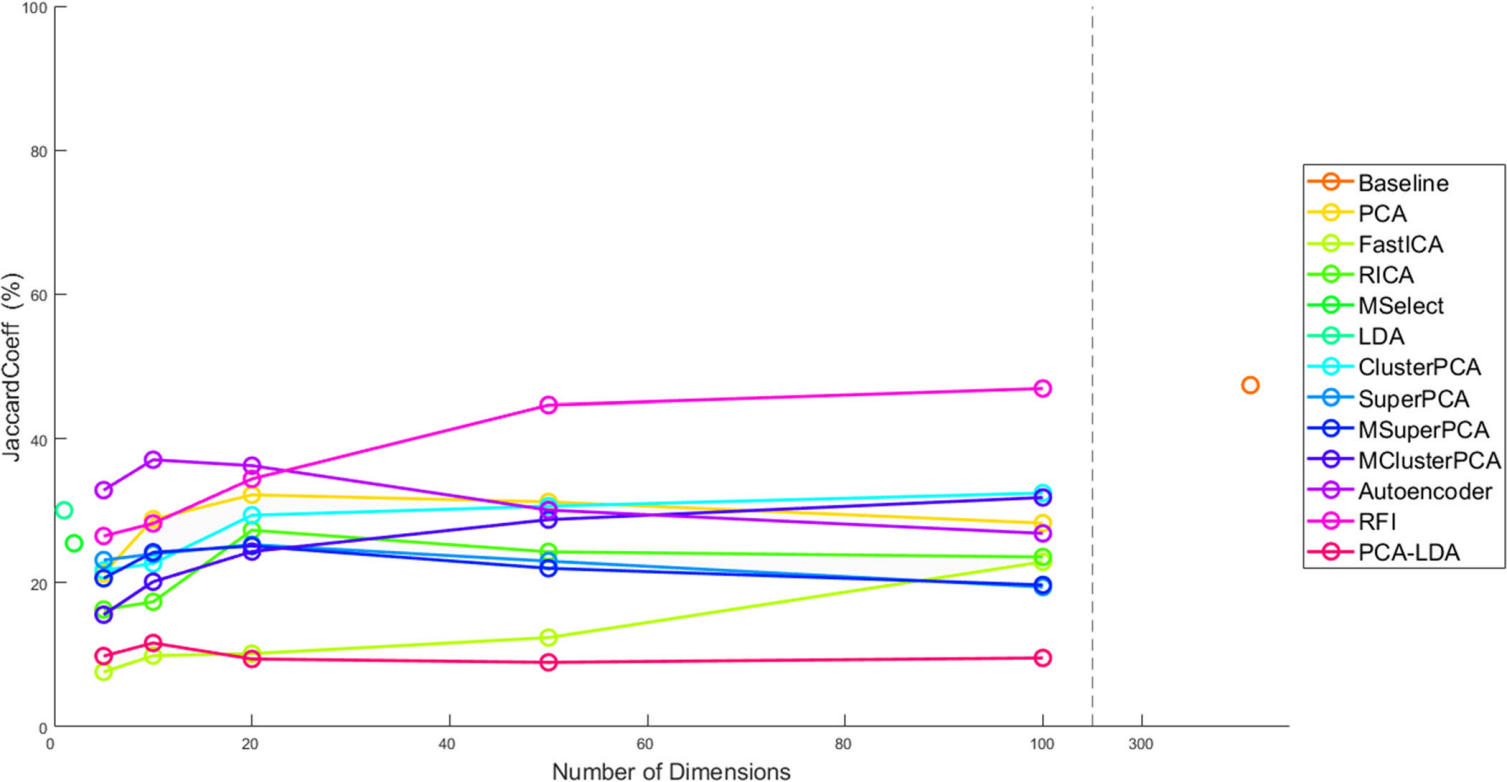
Comparison of fivefold validated Jaccard coefficient with respect to the number of retained dimensions

**FIGURE 5:**
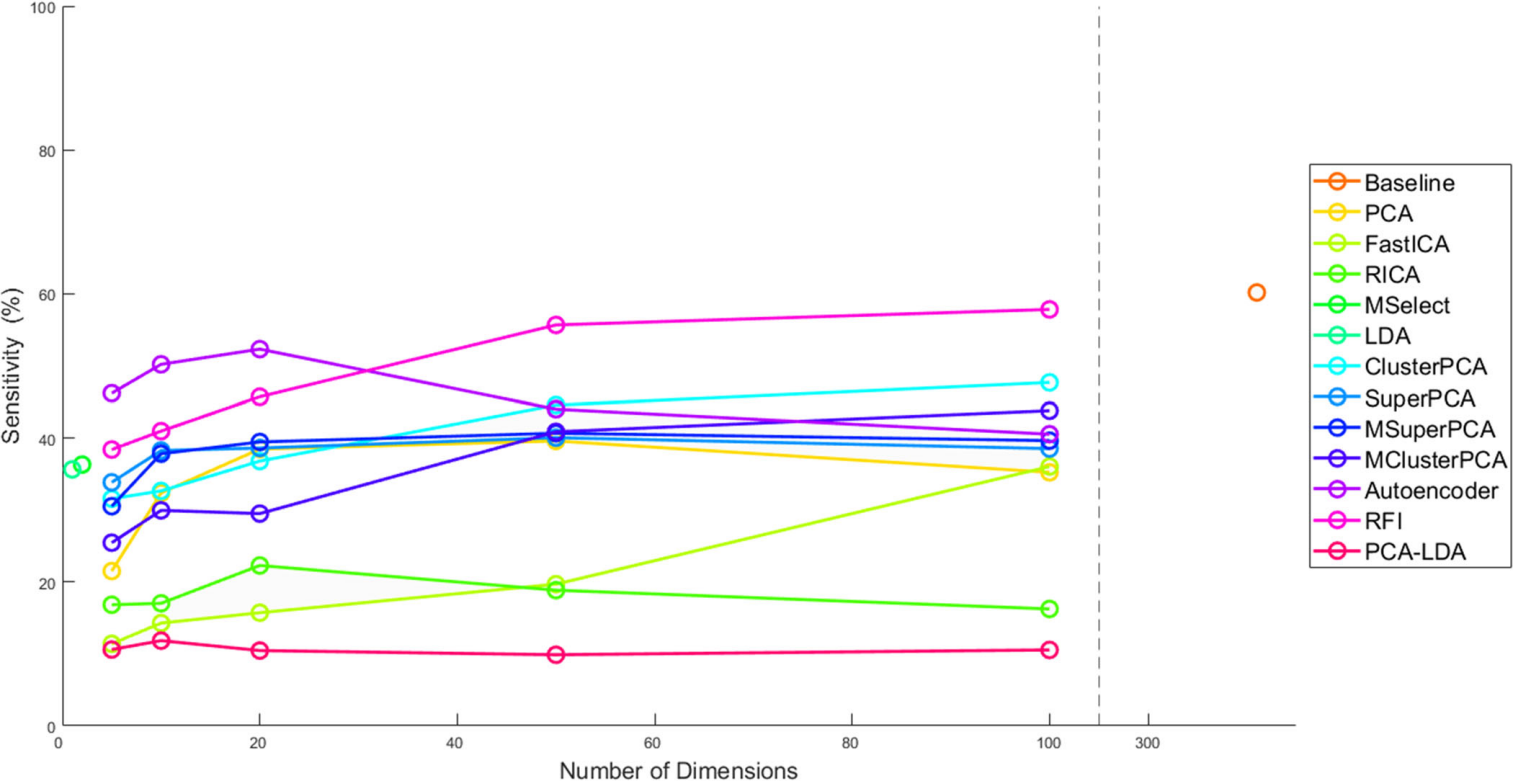
Comparison of fivefold validated sensitivity with respect to the number of retained dimensions

Each dimension reduction method was used to train an SVM classifier. The evolution of the fivefold cross‐validated JacCoef against the number of retained dimensions is demonstrated in Figure 4. The highest cross‐validated JacCoef was achieved for data input with reduction scheme: Baseline (no dimension reduction), RFI, and AE. Figure 4 shows that JacCoef drops after 20 dimensions for PCA, SuperPCA, MSuperPCA, RICA, and AE. For RFI and ClusterPCA, performance improves for a higher number of retained dimensions, reaching a maximum at 100 features. Although MSuperPCA is expected to enhance SuperPCA, this does not hold in this experiment and neither holds for ClusterPCA and MClusterPCA. MClusterPCA performs worse than ClusterPCA. This could be a sign that increasing the number of clusters trivializes the problem of dimension reduction, which in turn affects performance. Unexpectedly, FastICA shows the worst segmentation performance. Cross‐validated sensitivity in Figure 5 shows similar trends as JacCoef.

For each method, the number of retained dimensions was selected as that which achieved the highest cross‐validated JacCoef. The best performing combinations during validation and their respective cross‐validated performance metrics are shown in Table 2. Values are presented as an average across folds accompanied by SEM. Baseline and RFI achieve the best JacCoef at 47% during validation. MClusterPCA achieves one of the lowest SEM for JacCoef, compared to other schemes. Sensitivity values show high SEM for the different folds of validation, whereas specificity is affected to a lesser degree. Although PCA and ClusterPCA achieve a similar JacCoef, ClusterPCA has higher sensitivity with lower SEM. RICA achieves simultaneously the best specificity and worse sensitivity, an indicator of overfitting. All schemes suffer from low sensitivity. However, an experimentation of training an SVM with a high penalty for false negatives failed to considerably improve performance.

**TABLE 2:** Segmentation performance after fivefold validation

Method	*N*	JacCoef^a^ (%)	Sensitivity^a^ (%)	Specificity^a^ (%)
Baseline	311	47.38 (2.10)	60.19 (4.84)	86.18 (1.37)
PCA	20	32.20 (2.25)	38.47 (6.10)	75.13 (3.04)
FastICA	100	22.90 (1.52)	36.04 (4.55)	71.96 (3.35)
RICA	20	27.29 (1.42)	22.29 (2.95)	93.69 (0.23)
MSelect	2	25.48 (1.10)	36.31 (5.38)	88.28 (0.96)
LDA	1	30.05 (1.56)	35.64 (1.73)	82.52 (1.43)
PCA‐LDA	10	11.63 (2.36)	11.84 (3.62)	95.05 (0.82)
ClusterPCA	100	**32.44 (2.13)**	47.69 (4.97)	76.27 (2.78)
MClusterPCA	100	31.83 (1.68)	43.80 (4.44)	80.35 (2.42)
SuperPCA	20	25.28 (2.30)	38.61 (5.46)	84.07 (1.05)
MSuperPCA	20	25.10 (2.56)	39.46 (6.06)	85.85 (1.00)
Autoencoder	10	**37.07 (2.31)**	50.20 (5.19)	85.69 (0.68)
RFI	100	**46.91 (2.77)**	57.83 (4.95)	85.70 (1.21)

Abbreviations: ClusterPCA, cluster‐wise principal component analysis; JacCoef, Jaccard coefficient; LDA, linear discriminant analysis; MClusterPCA, multiscale cluster‐wise principal component analysis; MSelect, manual selection; RICA, reconstructed independent component analysis; RFI, random forest importance. *N* denotes the reduced dimension.

^a^Values are reported as mean(SEM).

**FIGURE 6:**
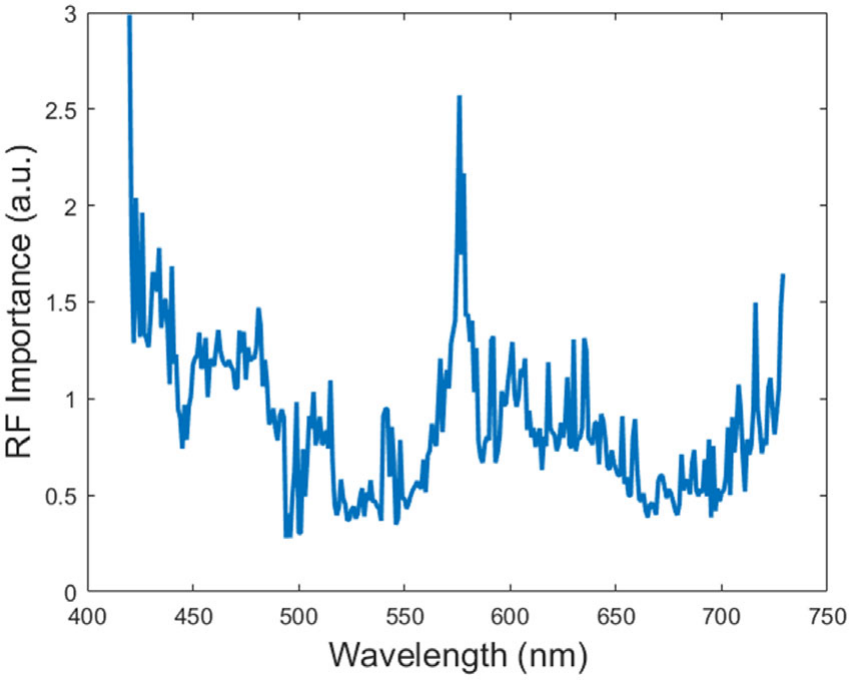
Random forest importance (RFI) importance values for each wavelength. Spectral measurements 430, 570, and 730 nm show the highest importance.

Because RFI performed best, we observed the types of features it extracted. RFI assigned the highest importance to bands at 570 and 730 nm, as shown in Figure 6. These coefficients are consistent with the pathophysiological properties of skin chromophores. RFI calculated an important coefficient at 420 nm, but this can be attributed to noise in that spectral band. The low limit of the range of the polarizers that were used in the HSI acquisition system is 420 nm.

**FIGURE 7:**
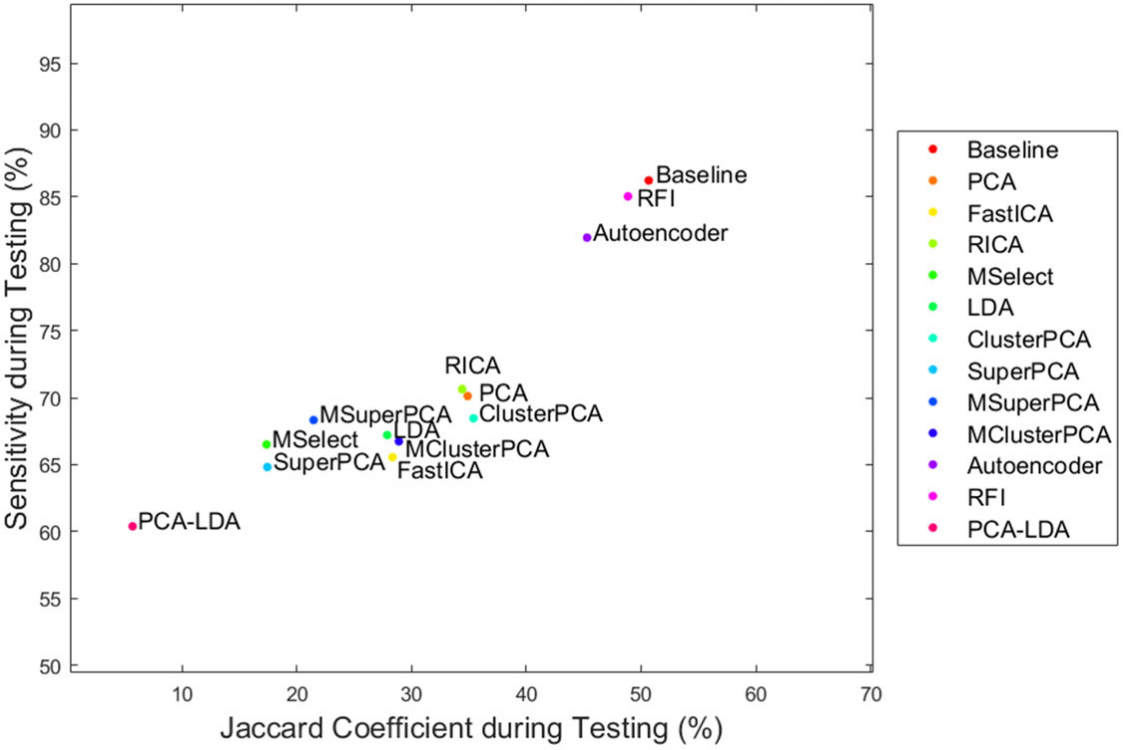
Testing performance in terms of Jaccard coefficient (*x*‐axis) and sensitivity (*y*‐axis) for the optimal classifiers during testing.

**FIGURE 8:**
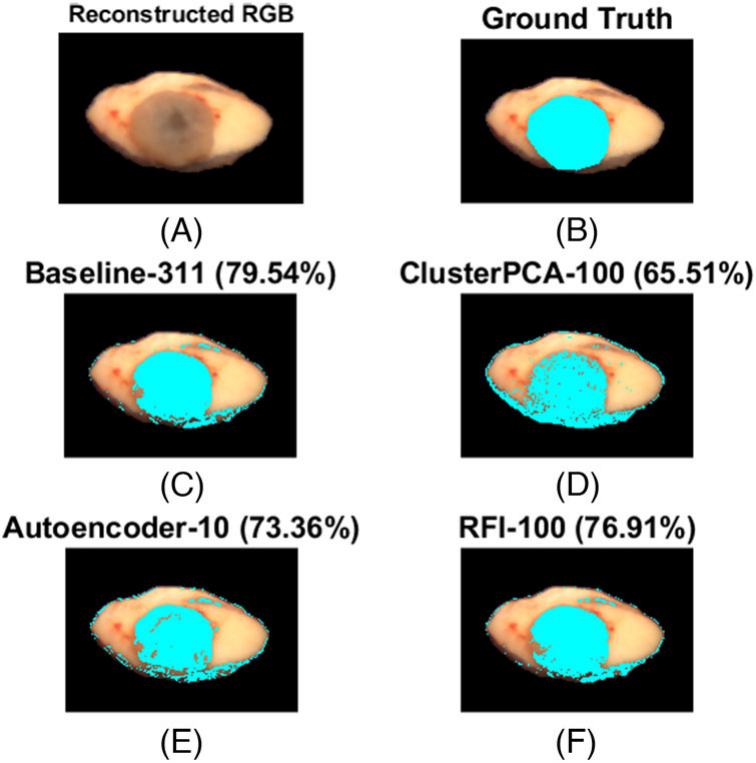
Results of tumor segmentation on an unknown test sample (melanocytic nevus) using pretrained classifiers. Subimage A shows the reconstructed RGB image, while subimages B‐F indicate tumor detection results using different dimension reduction methods and for a specific number of kept dimensions. Cyan pixels demonstrate true labels (ground truth) and predicted labels (baseline, cluster‐wise principal component analysis [ClusterPCA], autoencoder [AE], random forest importance [RFI]) for malignancy pixels. The number next to each method indicates the number of retained dimensions. The percentage value refers to the Jaccard coefficient.

**FIGURE 9:**
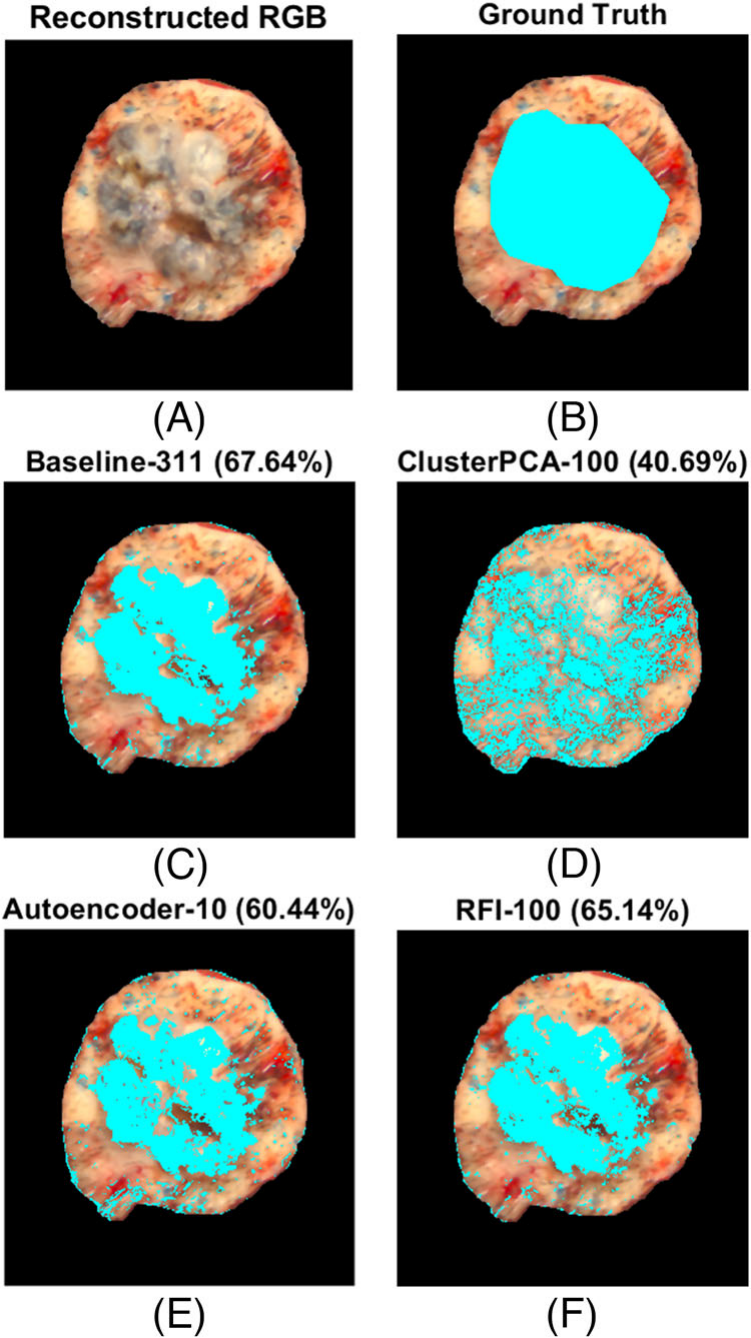
Results of tumor segmentation on an unknown test sample (basal cell carcinoma) using pretrained classifiers. Subimage A shows the reconstructed RGB image, while subimages B‐F indicate tumor detection results using different dimension reduction methods and for a specific number of kept dimensions. Cyan pixels demonstrate true labels (ground truth) and predicted labels (baseline, cluster‐wise principal component analysis [ClusterPCA], autoencoder [AE], random forest importance [RFI]) for malignancy pixels. The number next to each method indicates the number of retained dimensions. The percentage value refers to the Jaccard coefficient.

Each classifier was trained again on the entire validation set and tested on three new samples (basal cell carcinoma, melanocytic nevus, dermatofibroma). Most classifiers (baseline, ClusterPCA with 100 dimensions, AE with 10 dimensions, RFI with 100 dimensions) overperformed compared to validation and showed higher sensitivity to malignant pixel detection Figure 7. Margin detection for an intradermal melanocytic nevus sample in Figure 8 showed good results. FastICA, SuperPCA, and MSuperPCA failed to detect any tumor pixels at all. Moreover, all configurations classified the bottom tissue borders as malignant. Additionally, all apart from ClusterPCA mistakenly segmented a dark mark on the top right part of the tissue. However, all classifiers detected malignant pixels at the same general region as the ground‐truth labels. For an unknown sample of basal cell carcinoma, all classifiers detected the tumor area relatively well, as shown in Figure 9. ClusterPCA severely overestimated the tumor area. Again, some pixels on the sample's border were mistakenly detected. Only RFI and baseline managed to detect a compact tumor segment, without salt and pepper regions. This fact indicates that the spatial relationships among pixels should be considered during classification. Alternatively, post‐processing of the prediction mask with morphological operators can improve performance.

However, the accuracy of some methods comes at a cost. Train times are vastly different depending on the scheme for the dimension reduction of the SVM's input. The higher the complexity of the dimension reduction method, the higher the required training time. RFI and AE need an exceptionally long time to calculate the dimension reduction coefficients. However, SVM convergence time is not proportional to preprocessing training time. For this reason, training an SVM with AE‐reduced input takes less time than training with PCA‐reduced input for the same number of dimensions.

## 4 DISCUSSION

In the first part of this study, the eigenvectors of different PCA variations were observed. This is important because PCA is a staple tool at the initial stages of signal processing. The first eigenvector is similar for all PCA implementations and approximates the skin tissue base component.^47^ For PCA trained per sample, SuperPCA, and ClusterPCA, the second eigenvector has a twin peak that matches the twin peak of hemoglobin absorption curve. For all variations, the third eigenvector shows a large dip at approximately 600 nm, which coincides with the wavelength after which melanin absorption becomes more pronounced than hemoglobin absorption. When evaluating the transformed scores of each component, ClusterPCA shows more targeted information, compared to the rest. Additionally, two pathologists (MH and TI) evaluated visually the first three principal components of PCA variations and RICA. They indicated that ClusterPCA produces components with meaningful biological, whereas RICA extracted mostly illumination effects or artifacts. Overall, the proposed ClusterPCA method transforms spectral information more effectively than other PCA variations.

Segmentation performance of the same classifier is affected by the number of retained dimensions, and by the choice of dimension reduction method for data preprocessing. RFI performed well both during cross‐validation and during testing. Unexpectedly, the non‐reduced baseline also performed well. Although inputs reduced by AE and ClusterPCA show good cross‐validated performance, they do not perform so well on an unknown sample. All classifiers showed reduced sensitivity in tumor segmentation. This phenomenon could be attributed to the small number of dataset images and the choice of the test sample. The margins of some pigmented skin lesions are difficult to discriminate even during medical practice. Moreover, the small dataset affects the generalization ability of the classifier, as seen by the relatively high SME values during cross‐validation. Using a larger training dataset and a more complex classification scheme could improve performance. However, an SVM might not be suitable for a larger dataset. Because of the nature of HSI, emphasis should be given to classifiers that are well adjusted for HSI.^6^ However, the difficulty of obtaining labeled medical datasets hinders the application of compressors or classifiers based on neural networks. This was further demonstrated by AE, which performed lower than expected.

Although the dataset was small, a few observations can be made about individual dimension reduction methods. ICA and LDA, both staple methods, showed poor results during cross‐validation and testing, respectively. SuperPCA is a technique tuned for HSI datasets but suffered from a high false positive rate. ClusterPCA performed better than both PCA and SuperPCA in terms of the JacCoef. The eigenvectors of ClusterPCA could be correlated to the absorption spectra of skin chromophores, which enhances the explainability of the transform. Performance of SuperPCA and ClusterPCA did not improve with multiscale analysis, in contrast to previous work.^11^ However, experimentation with the hyperparameters of ClusterPCA, such as alternative spectral similarity measures and different number of endmembers, could potentially improve performance.

Generally, dimension reduction is applied on a per‐pixel basis. However, the advantage of snapshot HSI over RGB and other imaging modalities is the simultaneous imaging of both spatial and spectral dimensions. By applying dimension reduction per pixel, the spatial relationships of pixels are ignored. SuperPCA and ClusterPCA that group relevant pixels before dimension reduction are also a step in that direction. Moreover, deep‐learning feature extractors for images could utilize the combined spatio–spectral information.^48^ Transfer learning is a viable option to accelerate model building for segmentation. Because most segmentation models are developed for and trained on RGB images, dimension reduction is required to reduce the input to at minimum three dimensions. Thus, the results of this study regarding the performance of each dimension reduction method are still relevant.

The present study has several limitations in terms of dataset size and frequency of each pathology. A larger dataset that contains other types of pigmented skin lesions, as well as a variety of benign pathologies, could improve generalization and segmentation performance. Moreover, dimension reduction should ideally be trained on a separate dataset. Afterward, the segmentation task can be validated and tested on another dataset. However, in this study dimension reduction and segmentation were trained on the same dataset. Only the testing dataset was separate. Furthermore, no color standardization or artificial removal of diagnostic dyes was performed.

Insights on dimension reduction for skin tissue could facilitate the development of HSI‐based systems for skin cancer margin detection. Consequently, the cost and duration of pathology operations could be reduced, as well as the workload on the pathology laboratory. Future work should focus on dimension reduction methods that are well suited for skin tissue HSI and that provide an estimate for an appropriate number of retained dimensions. Furthermore, great care should be taken to make sure that rarely appearing features do not get discarded due to low variance or get overshadowed by system noise. To this end, we aim to evaluate a more targeted selection of dimension reduction methods on an enhanced dataset, using different machine learning and deep learning classifiers.

## 5 CONCLUSION

The choice of dimension reduction scheme and retained dimensions affects HSI‐based malignancy classification during skin gross pathology. Although some methods can extract features relevant to the absorbance of skin chromophores, the final classification suffers from low sensitivity and low generalization. Further investigation of dimension reduction methods suitable for skin tissue HSI is necessary.

## CONFLICTS OF INTEREST

The authors declare no conflicts of interest.

## Data Availability

The data that support the findings of this study are available on request from the corresponding author. The data are not publicly available due to privacy or ethical restrictions.
